# Single-phase full-bridge inverter control based on discrete adaptive sliding mode algorithm with error compensation

**DOI:** 10.1371/journal.pone.0334233

**Published:** 2025-10-10

**Authors:** Yun Zhang, Zhenyu Tang, Fenghui Xu, Kaichen Zhou, Kun Yang

**Affiliations:** 1 School of Rail Transportation, Shandong Jiao Tong University, Jinan, China; 2 Degree Programs in Systems and Information Engineering, University of Tsukuba, Tsukuba, Ibaraki, Japan; 3 Jinan JIMEILE Power Technology Co., Ltd, Jinan, China; University of Shanghai for Science and Technology, CHINA

## Abstract

This paper proposes that the control process of the single-phase full bridge inverter circuit is equivalent to two buck circuits, and the control strategy of the DC-DC circuit is adopted to enable the output voltage to track the given sine wave target value in real time, realizing the control of the inverter circuit, simplifying the control process, and enhancing the anti-interference ability of the system. On the basis of traditional discrete sliding mode control, a new adaptive approach rate is introduced, which can dynamically adjust the control gain according to the distance between the sliding surface and the sliding band. When the state variable is far from the sliding surface, it accelerates the approach speed, and when the state variable approaches the sliding surface, it reduces the approach speed, which can effectively reduce chattering. As a result, the width level of the sliding mode band is reduced from the traditional *O*(*T*) to the same level *O* (*T*^3^), and the width of the sliding mode band is significantly reduced, significantly improving the control accuracy and jitter suppression ability. The proposed control method was rigorously mathematically proven in terms of sliding mode bandwidth, jitter range, and convergence steps, and the advantages of the improved method in voltage tracking speed, steady-state error, and disturbance rejection performance were verified through multiple simulation experiments.

## 1. Introduction

With the development of energy technology, power electronic conversion technology has become increasingly important in industrial production. In the field of single-phase inverter power supplies, the system is required to output a stable sinusoidal AC voltage with good anti-interference capability. Traditional inverter power supplies generally adopt a voltage current dual closed-loop control strategy, which samples the output voltage and current in real time, calculates their effective values separately, and uses them as feedback signals for the current inner loop and voltage outer loop to achieve inverter control. This method is relatively complex, and the feedback signal is based on the effective value of the output AC signal, which has a certain lag and poor resistance to load disturbances. This article proposes that the control process of the single-phase full bridge inverter circuit is equivalent to two buck circuits, and the control strategy of the DC-DC circuit is adopted to enable the output voltage to track the given sine wave target value in real time, realizing the control of the inverter circuit, simplifying the control process, and enhancing the anti-interference ability of the system.

At present, DC-DC converters are widely used in motor drive [1], DC power supply and other occasions [2,3]. During the operation of DC-DC converters, disturbances caused by load changes can affect the output accuracy of the power supply [4]. It is necessary to study corresponding control algorithms to improve the system’s anti-interference performance and enhance its robustness. Scholars have proposed various control strategies for the stability and disturbance rejection performance of BUCK circuits, such as passive control [5], model predictive control [6], robust control [7], adaptive control [8], sliding mode control, and so on. Among them, sliding mode variable structure control has strong robustness to unknown disturbances and good control effects on nonlinear systems [9], and has been widely applied in various control systems. In practical engineering applications, to control power switching devices (such as MOSFETs, IGBTs, etc.) through digital controllers (such as DSP) to achieve energy conversion, it is necessary to design discrete digital control systems or discretize continuous control systems [10]. There are two main methods for designing discrete sliding mode controllers [11]. The first method is to discretize the system and design the controller; The second method is to design controllers for continuous systems and then discretize them. Due to the possibility that the second method may lead to a decrease in system stability [12], this paper adopts the first method to design a sliding mode controller for discrete systems. References [13,14] proposed a new discrete sliding mode reaching law that enables the system to enter the sliding mode band within a finite number of steps and cannot escape. Reference [13] proposed that the magnitude of sliding mode bandwidth is affected by external disturbances. To address the impact of external disturbances on system stability, references [15,16] proposed using delay estimation to estimate external disturbances and perform error compensation, thereby improving the robustness of the system. In addition, a disturbance-compensated sliding mode control (SMC) method has been proposed [17], where observer and feedforward structures were incorporated for applications in power electronic systems. For instance, in PMSM speed control, a novel SMC strategy was developed in which improved genetic algorithms were employed to optimize parameters, and a disturbance observer was designed to enhance robustness against load torque variations and parameter uncertainties. In the design process of the buck circuit controller, the coefficients before the selection function of the sliding mode controller will affect the time for the system to enter steady state. The larger the coefficient, the shorter the time required to enter steady state. On the other hand, excessively large coefficients will lead to an increase in the width of the sliding mode band, resulting in an increase in system jitter and a decrease in control accuracy.

This paper decomposes a single-phase full bridge inverter circuit into two buck circuits with positive and negative output voltages. The target waveform of the output voltage is set as a sine wave, and the output of single-phase AC power is achieved through real-time tracking control of the waveform, which has strong tracking and anti-interference capabilities; For the dynamic mathematical model of the buck circuit, a sliding mode control strategy is adopted to study the traditional discrete sliding mode control, traditional discrete adaptive sliding mode control, and discrete adaptive sliding mode control algorithms under error compensation. Stability proof and simulation analysis are conducted; Finally, experimental testing was conducted on the hardware platform to verify the correctness of the proposed control strategy.

Based on the discussion and analysis above, the main contributions of this study are as follows:

1. Equivalent the single-phase full bridge DC-AC inverter circuit to two BUCK circuits with positive and negative output voltages, transform the DC-AC problem into a DC-DC problem, set the target waveform of the output voltage as a sine wave, and achieve the output of single-phase AC power through real-time tracking of the sine waveform, improving control accuracy, enhancing real-time performance and anti-interference ability.
2. On the basis of traditional discrete sliding mode control, a new adaptive approach rate is introduced, which can dynamically adjust the control gain according to the distance between the sliding surface and the sliding band. When the state variable is far from the sliding surface, it accelerates the approach speed, and when the state variable approaches the sliding surface, it reduces the approach speed, which can effectively reduce chattering. As a result, the width level of the sliding mode band is reduced from the traditional O (*T*) to the same level O (*T*^3^), and the width of the sliding mode band is significantly reduced, significantly improving the control accuracy and jitter suppression ability.
3. The proposed control method was rigorously mathematically proven in terms of sliding mode bandwidth, jitter range, and convergence steps, and the advantages of the improved method in voltage tracking speed, steady-state error, and disturbance rejection performance were verified through multiple simulation experiments.

## 2. Principle and dynamic model of single-phase full bridge DC-AC conversion circuit

**Fig 1:**
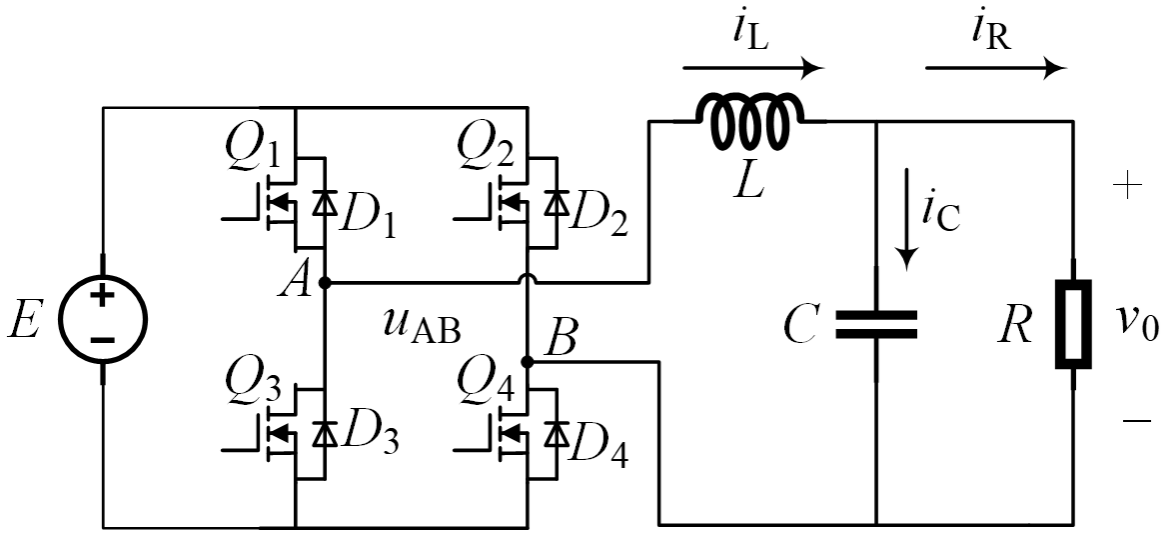
Single phase DC-AC circuit structure.

The topology structure of a single-phase full bridge DC/AC conversion circuit is shown in Fig 1, consisting of power supply *E*, inductor *L*, capacitor *C*, resistor *R*, and four power switching devices *Q*_1_ ~ *Q*_4_, where *D*_1_ ~ *D*_4_ are parasitic diodes of *Q*_1_ ~ *Q*_4_, respectively.

**Fig 2:**
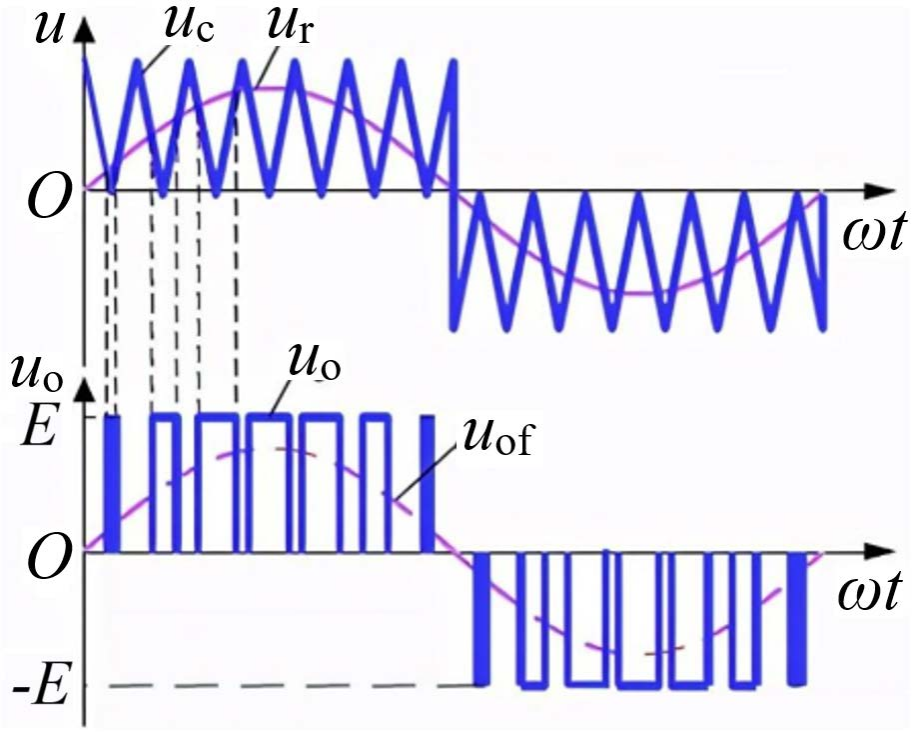
Unipolar SPWM control method.

The unipolar SPWM control method is adopted as shown in Fig 2. The modulation signal *u*_r_ is a sine wave, and the carrier signal *u*_c_ is a positive triangular wave in the positive half cycle of *u*_r_ and a negative triangular wave in the negative half cycle of *u*_r_.

**Fig 3:**
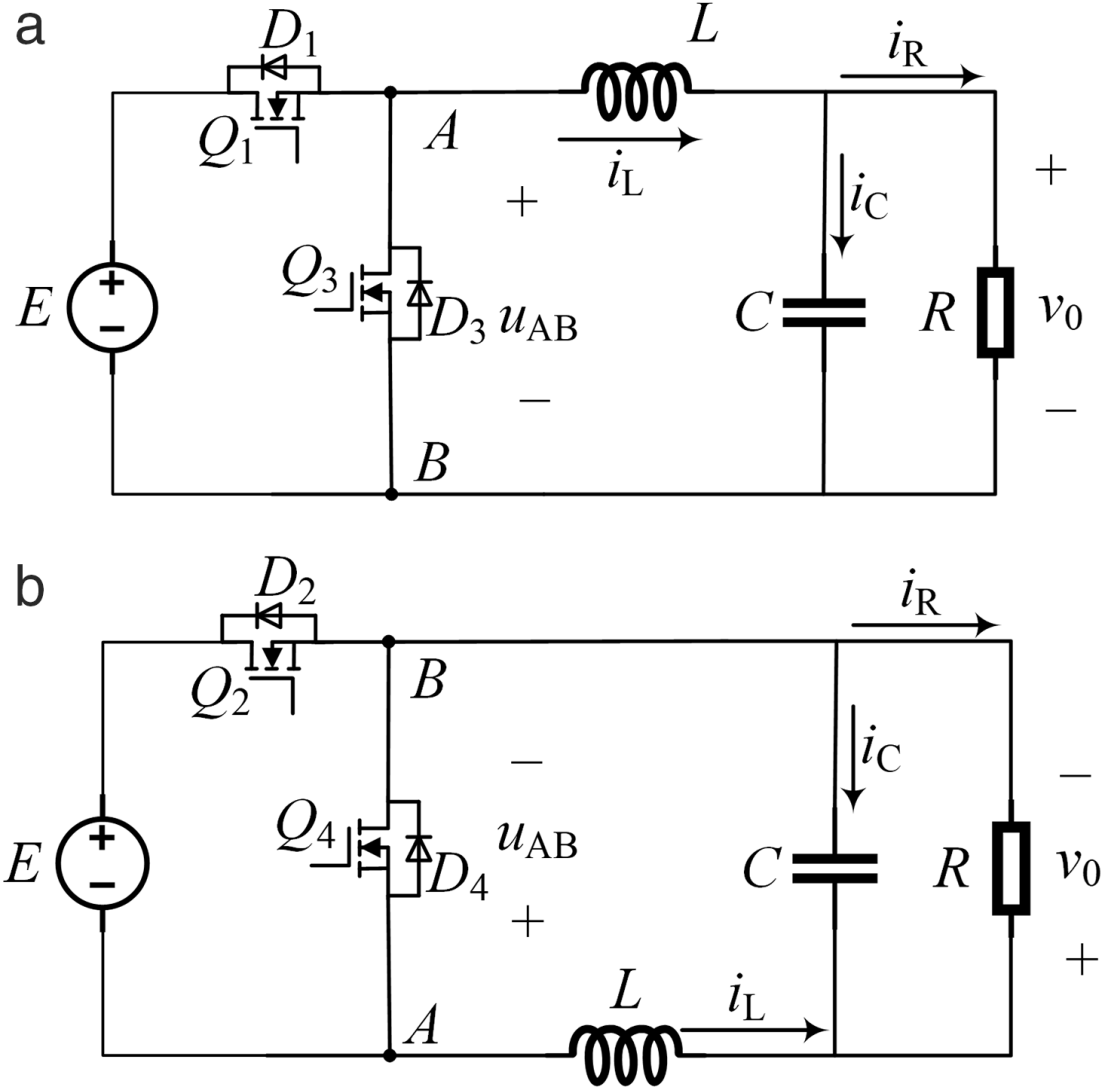
Equivalent circuit of unipolar positive and negative half cycle chopper. (a) The equivalent circuit of the positive half cycle of *u*_r_. (b) The equivalent circuit of the negative half cycle of *u*_r_.

In the positive half cycle of *u*_r_, *Q*_4_ remains conducting and *Q*_2_ remains off. When *u*_r _> *u*_c_, *Q*_1_ is turned on and *Q*_3_ is turned off, and in Fig 1, *u*_AB_ = *E*. When *u*_r_ ≤ *u*_c_, *Q*_1_ is turned off and *Q*_3_ is turned on, then *u*_AB _= 0. At this time, since *Q*_4_ remains conducting, it can be regarded as a short circuit, and *Q*_2_ remains off, which can be regarded as an open circuit. Fig 1 can be simplified to the equivalent circuit shown in Fig 3a. In the negative half cycle of *u*_r_, *Q*_1_ is turned off and *Q*_3_ remains conducting. When *u*_r _< *u*_c_, *Q*_2_ is turned on and *Q*_4_ is turned off, and *u*_AB_ =−*E*. When *u*_r _≥ *u*_c_, *Q*_2_ is turned off and *Q*_4_ is turned on, then *u*_AB _= 0. At this time, since *Q*_3_ remains conducting, it can be regarded as a short circuit, and *Q*_1_ remains off, which can be regarded as an open circuit. The circuit shown in Fig 1 is simplified to the equivalent circuit shown in Fig 3b, and the output voltage is negative at this moment.

**Fig 4:**
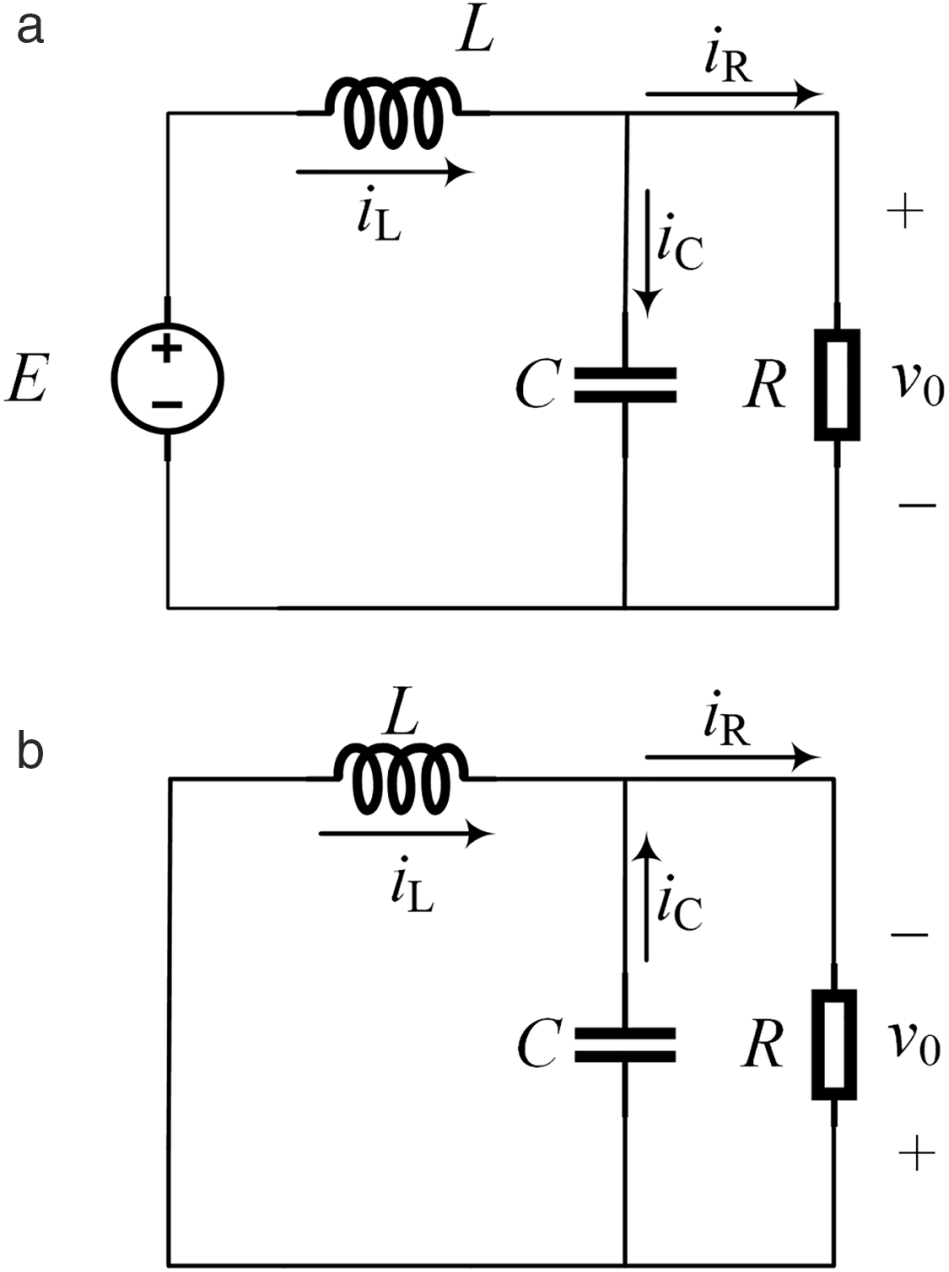
Working process of BUCK circuit. (a) Circuit structure with Q1 on and Q3 off. (b) Circuit structure with Q1 off and Q3 on.


The equivalent circuit in Fig 3 is a typical BUCK step-down circuit. Equivalent the DC/AC conversion circuit in Fig 1 to a simple DC/DC conversion circuit can simplify the control process. During the control process, the target value of the output voltage of the circuit in Fig 3a is set as the positive half cycle of the sine wave, and the target value of the output voltage of the circuit in Fig 3b is set as the negative half cycle of the sine wave. Finally, a sinusoidal alternating voltage is output to achieve the DC-AC conversion. In one control cycle of unipolar SPWM, the value range of the duty cycle *D* in Fig 3a is [0, 1], and the value range of the duty cycle *D* in Fig 3b is [−1, 0). Taking Fig 3a as an example, when *u*_r _> *u*_c_, turn on *Q*_1_ and turn off *Q*_3_. The circuit is shown in Fig 4a, and its mathematical model is shown in Equation (1). When *u*_r_ ≤ *u*_c_, turn on *Q*_3_ and turn off *Q*_1_. The circuit is shown in Fig 4b, and its mathematical model is shown in Equation (2).

$$\{\begin{array}{c} {}*20cC\frac{dv_{o}}{dt}=i_{L}-\frac{v_{o}}{R}\\ L\frac{di_{L}}{dt}=E-v_{o} \end{array}$$ (1)

$$\{\begin{array}{c} {}*20cC\frac{dv_{o}}{dt}=i_{L}-\frac{v_{o}}{R}\\ L\frac{di_{L}}{dt}=-v_{o} \end{array}$$ (2)

Let *v*_in_=μE, where *u* is the control ratio (i.e., the duty cycle *D*). According to the state-space averaging method, by combining Equations (1) and (2), the mathematical model of the single-phase full-bridge inverter circuit is obtained:


$$\left\{ \begin{array}{c} {\dot{v}}_{o}=\frac{i_{L}}{C}-\frac{v_{o}}{RC}\\ {\dot{i}}_{L}=\frac{uE}{L}-\frac{v_{o}}{L} \end{array}\right.$$ (3)

where *i*_L_ is the inductor current, *v*_o_ is the output voltage, *u* is the control ratio, and its value range is [−1, 1], which is used to generate the driving signals of the power transistors *Q*_1_ ~ *Q*_4_. Let *v*_r_ be the desired output voltage, then the output error *e*_1_ can be expressed as:


$$e_{1}=v_{o}-v_{r}$$ (4)

The derivative of the error *e*_1_ is:


$${\dot{e}}_{1}=\frac{i_{L}-i_{o}}{C}-{\dot{v}}_{r}$$ (5)

For the load disturbance during the operation of the power supply, Equation (5) can be rewritten in the form of Equation (6).


$$\begin{array}{c} {\dot{e}}_{1}=\frac{i_{L}-[i_{o}+(i-i_{o})]}{C}-{\dot{v}}_{r}\\ =\frac{i_{L}}{C}-\frac{v_{o}}{R_{o}C}-{\dot{v}}_{r}+\frac{v_{o}}{R_{o}C}-\frac{v_{o}}{RC}\\ =\frac{i_{L}}{C}-\frac{v_{o}}{R_{o}C}-{\dot{v}}_{r}+d \end{array}$$ (6)

In Equation (6), *i* represents the actual load current value, (*i*-*i*_o_) represents the additional load current caused by the load disturbance, *R*_o_ is the theoretical load resistance, *R* is the actual load resistance. Finally, the external disturbance is organized as:


$$d\left(t\right)=\frac{v_{o}}{R_{o}C}-\frac{v_{o}}{RC}$$

Let $e_{2}={\dot{e}}_{1}$. Differentiate *e*_*2*_ and combine it with Equation (3) to obtain Equation (7):


$$\begin{array}{c} {\dot{e}}_{2}=\frac{{\dot{i}}_{L}}{C}-\frac{{\dot{v}}_{o}}{R_{o}C}-{\ddot{v}}_{r}\\ =\frac{uE}{CL}-\frac{e_{1}}{CL}-\frac{v_{r}}{CL}-\frac{e_{2}}{R_{o}C}-\frac{{\dot{v}}_{r}}{R_{o}C}-{\ddot{v}}_{r}-\frac{d}{R_{o}C} \end{array}$$ (7)

According to the above analysis, the dynamic model of the system can be expressed as:


$$\left\{ \begin{array}{c} {\dot{e}}_{1}=e_{2}+d_{1}\\ {\dot{e}}_{2}=\frac{uE}{CL}-\frac{e_{1}}{CL}-\frac{v_{r}}{CL}-\frac{e_{2}}{R_{o}C}-\frac{{\dot{v}}_{r}}{R_{o}C}-{\ddot{v}}_{r}-\frac{d}{R_{o}C} \end{array}\right.$$ (8)

Generally, the continuous-time system with external disturbances is expressed as Equation (7):


$$\dot{x}=Ax+Bu+Df$$ (9)

In Equation (9), *x* ∈ *R*^n^ is the state vector, *u* ∈ *R*^m^ is the control input, and *f* ∈ *R*^m^ is the external disturbance. In an actual power supply system, the condition that the disturbance is bounded is generally satisfied. Let the desired voltage *v*_r_ be a constant, and its derivatives of all orders are 0. By combining Equations (3) and (8), the state equation of the system is:


$$\left[\begin{array}{c} {}*20c{\dot{i}}_{L}\\ {\dot{v}}_{o}\\ {\dot{e}}_{1}\\ {\dot{e}}_{2} \end{array}\right]=\left[\begin{array}{cccc} {}*20c0 & -\frac{\text{1}}{L} & 0 & 0\\ \frac{\text{1}}{C} & -\frac{\text{1}}{RC} & 0 & 0\\ \frac{\text{1}}{C} & -\frac{\text{1}}{R_{o}C} & 0 & 0\\ 0 & -\frac{\text{1}}{LC} & 0 & -\frac{\text{1}}{R_{o}C} \end{array}\right]\left[\begin{array}{c} {}*20ci_{L}\\ v_{o}\\ e_{1}\\ e_{2} \end{array}\right]+\left[\begin{array}{c} {}*20c\frac{E}{L}\\ 0\\ 0\\ \frac{E}{LC} \end{array}\right]u+\left[\begin{array}{c} {}*20c0\\ 0\\ d\\ \frac{d}{R_{o}C} \end{array}\right]$$ (10)

## 3. Design of adaptive sliding mode control (SMC) rate under discrete-time system

Using a zero order keeper (ZOH) to convert the controller *u*(*t*) into discrete form, expressed as *u*(*k*)=*u*(*t*), *k*∈[*kT,*(*k* + 1)*T*], where *T* is the sampling period, Equation (11) is obtained:


$$x_{k+1}=\Phi x_{k}+\Gamma u_{k}+d_{k}$$ (11)

Define *x*_k_ in the discrete state as *x*(*kT*), *u*_k_ as *u*_k_(*kT*), *d*_k_ as *d*_k_(*kT*), $d_{k}=\int_{0}^{T}e^{A\tau }Df((k+1)T-\tau )d\tau$, Γ=$\int_{0}^{T}e^{A\tau }d\tau B$, Φ = *e*^*AT*^. And assume that *d*_*k*_ is bounded.

*Remark* 1: The external disturbance matrix *d*_*k*_ of the system has the following characteristics [18,19], *d*_*k*_ = *O*(*T*), *d*_*k*_-*d*_*k*-1_ = *O*(*T*^2^), (*d*_*k*_-*d*_*k-*1_)-(*d*_*k*_-_1_-*d*_*k*-2_)=*O*(*T*^3^). Moreover, the range of the external disturbance *d* is bounded, that is, |*d*| ≤ d^*^, d^*^ = *O*(*T*), and the external disturbance matrix *d*_*k*_ is bounded, |*d*_*k*_| ≤ *d*_k_^*^.

According to the forward Euler method, the discrete error expression is shown in Equation (12), where *T* is the sampling period:


$$\left\{ \begin{array}{c} e_{1}(k+1)=e_{1}(k)+Te_{2}(k)+Td_{1}(k)\\ e_{2}(k+1)=e_{2}(k)+T(\frac{uE}{CL}-\frac{v_{o}}{CL}-\frac{e_{2}}{R_{o}C}+\frac{d}{R_{o}C}) \end{array}\right.$$ (12)

For the adaptive sliding mode control system of converter (8), where 0 < *Tc*_1_ < 1, its sliding surface can be designed as follows:


$$s_{k}=Cx_{k}=\left[\begin{array}{cccc} {}*20c0 & 0 & c_{1} & 1 \end{array}\right]\left[\begin{array}{c} {}*20ci_{L}\\ v_{o}\\ e_{1}\\ e_{2} \end{array}\right]$$ (13)

Among them, *C* ∈ *R*^1×n^ and *C*Γ ≠ 0. Design a reasonable sliding surface to enable the state variable to move from any initial state to 0 in a finite time, i.e.,:


$$S=\{x_{k}|Cx_{k}=0\}$$ (14)

*Remark* 2: Let *ξ*_*k *_= *C*(*d*_*k*_-2*d*_*k*-1_ + *d*_*k*-2_), then *ξ*_*k*_ ∈ *O*(*T*^3^). And assume that the rate of change of the error *f*_*k*_ is bounded:


$$|\xi_{k}|\leq \xi$$ (15)

*Theorem* 1: If the system (9) is stable, then the state variable will eventually move into the sliding mode band, that is, for any *k *> k^*^, the *s*_*k*_ satisfies Equation (16):


$$\left|s_{k}|\leq \Delta_{i},(i=1,2\right)$$ (16)

*Theorem* 2: If the sliding mode surface *s*_*k*_ satisfies the following conditions, the state variables will eventually enter the sliding mode band and reach a stable state:


$$\left\{ \begin{array}{c} \left|s_{k+1}|<|s_{k}|,if|s_{k}|>\Delta_{i}(i=1,2\right)\\ \left|s_{k+1}|\leq \Delta_{i},if|s_{k}|\leq \Delta_{i}(i=1,2\right) \end{array}\right.$$ (17)

*Lemma* 1: Consider a dynamic system [20]:


y(k+1)=y(k)−ly(k)+g(k) (18)

If |*l*| < 1 and |*g*(*k*)| < *γ*, where *γ* > 0, then *y*(*k*) is always bounded, and its range is |*y*(*k*)| <* γ*/|*l*|.

### 3.1. The traditional discrete sliding mode control system

Substitute the mathematical model of Equations (8) into (13), and solve it by using the equilibrium state control:


s(k+1)=0 (19)

The Equation (20) can be obtained:


$$c_{1}(e_{1}(k)+Te_{2}(k)+Td(k))+e_{2}(k)+T\frac{E}{CL}u-T\frac{v_{o}}{CL}-T\frac{e_{2}}{R_{o}C}+\frac{Td}{R_{o}C}=0$$ (20)

Furthermore, the controller can be obtained as:


$$u=-\frac{CL}{ET}\{c_{1}[e_{1}(k)+Te_{2}(k)]+e_{2}(k)-\frac{Tv_{o}}{CL}-\frac{Te_{2}}{R_{o}C}\}$$ (21)

Under the controller (21), the final state of the sliding mode surface is:


$$s(k+1)=c_{1}Td(k)+\frac{Td}{R_{o}C}$$ (22)

When the system satisfies the conditions of *Remark* 1, the range of the sliding mode band is:


$$|s(k)|\leq c_{1}Td^{*}+\frac{Td^{*}}{R_{o}C}$$ (23)

The following discusses the dynamic performance of the error *e*_1_ under the control of the traditional discrete sliding mode controller (21) as follows:


$$e_{1}(k+1)=e_{1}(k)-Tc_{1}e_{1}(k)+T(s(k)+d(k))$$ (24)

The value range of *e*_1_ is as follows:


$$|e_{1}(k)|\leq \frac{T|c_{1}Td^{*}+\frac{Td^{*}}{R_{o}C}+d^{*}|}{Tc_{1}}=Td^{*}+\frac{Td^{*}}{R_{o}Cc_{1}}+\frac{d^{*}}{c_{1}}$$ (25)

*Remark* 3: According to Equation (21), the magnitude of the width of the sliding mode band mainly depends on *Td*/*R*_o_*C*. In *Remark* 1, *d*_*k*_ = *O*(*T*). Here, *R*_o_C is a number less than 1. Therefore, the magnitude of the width of the sliding mode band is less than *O*(*T*).

According to Equation (23), no matter how small the sampling period *T* is, the influence of the external disturbance in Equation (25) on the system cannot be reduced. Therefore, the traditional discrete sliding mode control system has poor ability to resist external disturbances. In order to reduce the width of the sliding mode band and enhance the robustness of the system, this paper proposes a discrete adaptive sliding mode control method.

### 3.2. The discrete adaptive SMC for the single-phase full-bridge DC/AC conversion system

Consider the following discrete adaptive SMC reaching law:


$$s_{k+1}=\alpha Cx_{k}-\frac{\delta }{\varphi (k)}sgn(Cx_{k})$$ (26)

where 0<*α*<1 and *δ*>*C*$d_{k}^{*}$ >*ξ*. And


$$\varphi (k)=\gamma +(1-\gamma )(|s_{k}|+1{)}^{-l}$$ (27)

where *l* > 0 and 0 < *γ* < 1:

Through the analysis of the reaching law (26), it can be known that:

(1) If |*s*_*k*_| increases, then the denominator of the second term in Equation (26) will tend to *γ*, and (*δ*/*γ*)> *δ*.
(2) If |*s*_*k*_| decreases, then *φ*(*k*) ≈ *γ*+(1-*γ*) =1, and *δ*/*φ*(*k*) tends to *δ*.

It can be known from the above analysis that when the state variable is far from the sliding mode band, the coefficient before the selection function *sgn* will increase, thereby accelerating the speed at which the system enters the sliding mode band and reaches the stable state. When the state variable approaches the sliding mode surface, the coefficient before the selection function *sgn* will decrease, and the function of suppressing the chattering will be finally achieved. The above process reflects the characteristics of the adaptive reaching law, and solves the problem that the influence of external disturbances on the error cannot be eliminated in the traditional discrete sliding mode control system, thus improving the robustness of the system.

Based on the above control method, and by combining Equations (11) and (26) to solve the equation *s*(*k* + 1) = 0, the controller can be obtained:


$$C(\Phi x_{k}+\Gamma u_{k}+d_{k})=\alpha {\left[\begin{array}{c} {}*20c0\\ 0\\ c_{1}\\ 1 \end{array}\right]}^{T}\left[\begin{array}{c} {}*20ci_{L}\\ v_{o}\\ e_{1}\\ e_{2} \end{array}\right]-\frac{\delta }{\varphi (k)}sgn({\left[\begin{array}{c} {}*20c0\\ 0\\ c_{1}\\ 1 \end{array}\right]}^{T}\left[\begin{array}{c} {}*20ci_{L}\\ v_{o}\\ e_{1}\\ e_{2} \end{array}\right])$$ (28)

Equation (28) satisfies the condition of bounded disturbance in *Remark* 2. However, due to the external disturbance cannot be measured, the controller is shown in Equation (29):


$$u_{k}=(C\Gamma {)}^{-1}(\alpha s_{k}-\frac{\delta }{\varphi (k)}sgn(s_{k})-C\Phi x_{k})$$ (29)

The sliding mode state expression of the dynamic system can be obtained:


$$s_{k+1}=\alpha s_{k}-\frac{\delta }{\varphi (k)}sgn(s_{k})+Cd_{k}$$ (30)

*Proposition 1*

1) The controller (27) will cause the system to monotonically enter the sliding surface, and the range of the sliding surface is

$$\theta =\{s_{k}||s_{k}|\leq \Delta_{1}=\frac{\delta }{\varphi }+Cd_{k}^{*}\}$$ (31)

2) The state variable will not escape after entering the sliding mode band
3) The order of magnitude of the sliding band width is *O*(*T*), which is Δ_1_ ∈* O*(*T*)

*Proof*

1) If the state variable is outside the sliding surface, then if |*s*_*k*_| > Δ_1_, then |*s*_*k*+1_| < |*s*_*k*_|:

When *s*_*k*_>Δ_1_>0, according to *Remark*1, $\left|d_{k}\right|\leq^{d_{k}^{*}}$ and *φ*∈(γ,1), $\delta >C^{d_{k}^{*}}>\xi$, then:


$$s_{k+1}=\alpha s_{k}-\frac{\delta }{\varphi (k)}sgn(s_{k})+Cd_{k}<s_{k}-\frac{\delta }{\varphi (k)}+Cd_{k}^{*}<s_{k}$$ (32)

When *s*_*k*_ < -Δ_1_ < 0, then:


$$s_{k+1}=\alpha s_{k}+\frac{\delta }{\varphi (k)}sgn(s_{k})+Cd_{k}>s_{k}$$ (33)

The above analysis shows that if *s*_*k*_ is outside the range of [-Δ_1_, Δ_1_], the state variables will continuously approach the sliding mode band, with a width of [-Δ_1_, Δ_1_].

2) When the state variable and sliding surface satisfy *s*_*k*_ = *Cx*_*k*_, the sliding surface is within the range of [-Δ_1_, Δ_1_]. Discuss the direction of the sliding surface when *s*_*k*_ is positive and negative, respectively.

When 0<*s*_*k*_<Δ_1_, since 0<*s*_*k*_, we have $sgn(s_{k})=1$ Therefore $s_{k+1}=\alpha s_{k}-\frac{\delta }{\varphi (k)}+Cd_{k}$, combining with (32) and (33), we obtain:


$$\begin{array}{c} s_{k+1}-(-\Delta_{1})=\alpha s_{k}-\frac{\delta }{\varphi (k)}+Cd_{k}+\frac{\delta }{\varphi }+Cd_{k}^{*}>0;\\ s_{k+1}-s_{k}=(\alpha -1)s_{k}-\frac{\delta }{\varphi (k)}+Cd_{k}<-\frac{\delta }{\varphi (k)}+Cd_{k}<0. \end{array}$$ (34)

From the above analysis, it can be concluded that -Δ_1_ < *s*_*k*+1_ < *s*_*k*_.

When -Δ_1_<*s*_*k*_<0, since *s*_*k*_ <0, we have $sgn(s_{k})=-1$ Therefore $s_{k+1}=\alpha s_{k}+\frac{\delta }{\varphi (k)}+Cd_{k}$, combining with (32) and (33), we obtain:


$$\begin{array}{c} s_{k+1}-\Delta_{1}=\alpha s_{k}+\frac{\delta }{\varphi (k)}+Cd_{k}-\frac{\delta }{\varphi }-Cd_{k}^{*}>0;\\ s_{k+1}-s_{k}=(\alpha -1)s_{k}+\frac{\delta }{\varphi (k)}+Cd_{k}>\frac{\delta }{\varphi (k)}+Cd_{k}<0. \end{array}$$ (35)

In summary, *s*_*k*+1_ < *s*_*k*_ < Δ_1_ is obtained. When the state variable enters the sliding mode band, i.e., -Δ_1_ < *s*_*k*_ < Δ_1_, then *s*_*k*+1_∈(-Δ_1_, Δ_1_), and the state variable will not escape the sliding mode band.

According to *Proposition* 1, *C*$d_{k}^{*}$∈ *O*(*T*), if the state variable enters the sliding mode band and tends to stabilize, it will tend towards *δ*, then Δ_1_≈*δ*+ *C*$d_{k}^{*}$. By selecting an appropriate *δ* so that the order of *δ* is the same as that of *C*$d_{k}^{*}$, the order of magnitude of the sliding mode bandwidth *O*(*T*)+*O*(*T*)=*O*(*T*) can be obtained.

*Proposition* 2: Regarding the traditional discrete adaptive sliding mode control method, the selection function *sgn* is used to reduce the interference of external disturbances on the system. Compared with the traditional sliding mode control method, the sliding band width of the discrete adaptive sliding mode control algorithm is narrower, and the control accuracy is improved. This paper proposes a discrete adaptive sliding mode control algorithm based on error compensation, which will further improve the control accuracy.

### 3.3. Discrete adaptive SMC with error compensation for the single-phase full-bridge DC/DA conversion system

For the discrete adaptive sliding mode control algorithm, a new approach rate representation method [11] is adopted:


$$s_{k+1}=\alpha Cx_{k}-\frac{\delta }{\varphi (k)}sgn(Cx_{k})+C(d_{k}-2d_{k-1}+d_{k-2})$$ (36)

This paper employs the time-delay estimation perturbation method to estimate load disturbances, and incorporates the estimated disturbance values into the controller, thereby enhancing the robustness and accuracy of the controlled system. According to Equation (12), the disturbance estimation can be obtained:


$$\hat{d}(k)=d(k-1)=\frac{e_{1}(k)-e_{1}(k-1)}{T}-e_{2}(k-1)$$ (37)

By substituting Equations (11) and (36) into the equation s(k + 1)=0, we can obtain the controller:


$$u_{k}=(C\Gamma {)}^{-1}\left[\alpha s_{k}-\frac{\delta }{\varphi (k)}sgn(s_{k})-C\Phi x_{k}-C(2d_{k-1}-d_{k-2})\right]$$ (38)

**Fig 5:**
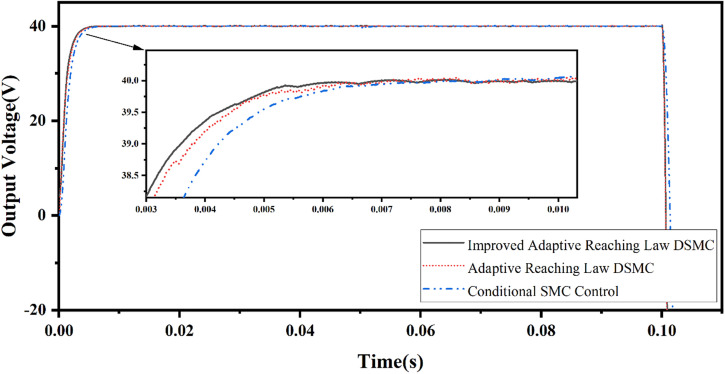
Program flowchart.

The program flowchart shown in Fig 5 illustrates the implementation procedure of the proposed controller in (38).

The flowchart summarizes the implementation steps of the controller in (38). The procedure includes sensor data acquisition, error calculation $e_{\text{1}}$, $e_{\text{2}}$, computation of $s_{k}$, solving the discrete matricesΦ,Γ, and external disturbance estimation d, followed by the calculation of the control input u and its application to the hardware platform.

*Proposition 2:*

1) The controller (38) can make the system monotonically enter the sliding surface, and the range of the sliding surface is:

$$\theta =\{s_{k}||s_{k}|\leq \Delta_{2}=\frac{\delta }{\varphi }+\xi \}$$ (39)

2) The state variable will not escape after entering the sliding mode belt.
3) The width order of the slip band is *O*(*T*^*3*^), that is Δ_2_ ∈* O*(*T*^*3*^).

*Proof:*

1) Refer to the proof process of proposition 1, and satisfy *φ*∈(γ,1), *δ* > *ξ*. The value of *s*_*k*_ is classified and discussed, and the following results can be obtained:

$$\left\{ \begin{array}{c} s_{k+1}=\alpha s_{k}-\frac{\delta }{\varphi (k)}sgn(s_{k})+C(d_{k}-2d_{k-1}+d_{k-2})<s_{k}(s_{k}>\Delta_{2}>0)\\ s_{k+1}=\alpha s_{k}+\frac{\delta }{\varphi (k)}sgn(s_{k})+C(d_{k}-2d_{k-1}+d_{k-2})>s_{k}(s_{k}<-\Delta_{2}<0) \end{array}\right.$$ (40)

It can be obtained that the sliding mode band width of the system is [-Δ_2_, Δ_2_] under the controller (38).

2) Referring to the proof process of proposition 1, the following results can also be obtained:

$$\left\{ \begin{array}{c} s_{k+1}-(-\Delta_{2})>0,s_{k+1}-s_{k}<0;(0<s_{k}<\Delta_{2})\\ s_{k+1}-\Delta_{2}>0,s_{k+1}-s_{k}<0;(-\Delta_{2}<s_{k}<0) \end{array}\right.$$ (41)

From the conclusion of comprehensive Equation (39), it can be seen that after the state variable enters the sliding mode band, that is, *s*_*k*_∈[-Δ_2_, Δ_2_], the state variable will never escape from the sliding mode band.

3) In remark 2, it is concluded that *ξ*_*k*_ ∈ *O*(*T*^3^), if the state variable enters the sliding mode band and reaches a stable state, $\frac{\delta }{\varphi (k)}$ will tend to *δ*, and finally Δ_2_≈(*δ* + *ξ*). By adjusting the value of *δ*, the magnitude of *δ* is approximate to *ξ* and finally the magnitude of sliding mode band width is *O*(*T*^3^)+ *O*(*T*^3^)= *O*(*T*^3^). By observing the above analysis results, it can be seen that when the disturbance estimation is added to the controller, the oscillation range of the sliding mode band is reduced, and the steady-state effect of the system is better.

*Proposition 3:*

Suppose that the system enters the sliding mode band through N^*^ steps under the control of the controller (36), then we will discuss the classification according to the state of *s*_*k*_ and calculate the number of operation steps N^*^.

*Proof:*

① When *s*_*k*_ > 0, *k* = 0, 1, 2…… according to (34):


$$\left\{ \begin{array}{c} s_{1}=\alpha s_{0}-\frac{\delta }{\varphi (0)}+\xi_{0}(k=0)\\ s_{2}=\alpha s_{1}-\frac{\delta }{\varphi (1)}+\xi_{1}\\ \;\;=\alpha^{2}s_{0}-\alpha \frac{\delta }{\varphi (0)}+\alpha \xi_{0}-\frac{\delta }{\varphi (1)}+\xi_{1}(k=1)\\ \;\;\vdots \\ s_{k}=\alpha^{k}s_{0}-\sum_{k=0}^{n-1}\alpha^{n-1-k}\frac{\delta }{\varphi (k)}+\sum_{k=0}^{n-1}\alpha^{n-1-k}\xi_{k}(k=n) \end{array}\right.$$ (42)

It can be seen from remark 2 that there is *ξ* ≥ *ξ*_*i*_ that (42) can be written as:


$$\begin{array}{c} s_{n}\leq \alpha^{k}s_{0}-\sum_{k=0}^{n-1}\alpha^{n-1-k}\frac{\delta }{\varphi (k)}+\sum_{k=0}^{n-1}\alpha^{n-1-k}\xi \\ \;=\alpha^{n}s_{0}-\sum_{k=0}^{n-1}\left[\frac{\text{1}}{\varphi (k)}-\frac{\xi }{\delta }\right]\alpha^{n-1-k}\delta \end{array}$$ (43)

From the recurrence formula of *s*_*k*_, it can be seen that when *s*_*k*_ > 0, $\sum_{k=0}^{n-1}\left[\frac{\text{1}}{\varphi (k)}-\frac{\xi }{\delta }\right]\alpha^{n-1-k}\delta$ will be subtracted from each operation until *s*_*k*_ enters the sliding mode band *s*_*k*_∈[-Δ_2_, Δ_2_], and finally reaches a stable state. This process reflects the characteristics of gradual stability of the system.

Suppose $\varphi^{\prime }$∈ (0, 1) exists to make the following equation true:


$$\sum_{k=0}^{n-1}\left[\frac{\text{1}}{\varphi (k)}-\frac{\xi }{\delta }\right]\alpha^{n-1-k}=\sum_{k=0}^{n-1}\left[\frac{\text{1}}{\varphi^{\prime }}-\frac{\xi }{\delta }\right]\alpha^{n-1-k}$$ (44)

Then the value of $\varphi^{\prime }$ can be calculated as:


$$\varphi^{\prime }=\frac{1-\alpha^{n}}{(1-\alpha^{n})\frac{\xi }{\delta }+(1-\alpha )\sum_{k=0}^{n-1}\left[\frac{\text{1}}{\varphi (k)}-\frac{\xi }{\delta }\right]\alpha^{n-1-k}}$$ (45)

When the right side of equation (43) is equal to zero, that is, *s*_*k*_ is equal to zero, and the system reaches steady state, n^*^ is:


$$n^{*}=\log_{\alpha }\frac{\left(\frac{\text{1}}{\varphi^{\prime }}-\frac{\xi }{\delta }\right)\delta }{(1-\alpha )s_{0}+\left(\frac{\text{1}}{\varphi^{\prime }}-\frac{\xi }{\delta }\right)\delta }$$ (46)

② When *s*_*k*_ < 0, *k* = 0, 1, 2……, the recurrence formula of *s*_*k*_ can be obtained by using a similar derivation method:


$$\begin{array}{c} s_{n}=\alpha^{k}s_{0}+\sum_{k=0}^{n-1}\alpha^{n-1-k}\frac{\delta }{\varphi (k)}+\sum_{k=0}^{n-1}\alpha^{n-1-k}\xi_{k}\\ \;\;\geq \alpha^{n}s_{0}-\sum_{k=0}^{n-1}\left[\frac{\text{1}}{\varphi (k)}-\frac{\xi }{\delta }\right]\alpha^{n-1-k}\delta \end{array}$$ (47)

When the right side of equation (47) is equal to zero, that is, *s*_*k*_ is equal to zero, and the system reaches steady state, n^*^ is:


$$n^{*}=\log_{\alpha }\frac{\left(\frac{\text{1}}{\varphi^{\prime }}-\frac{\xi }{\delta }\right)\delta }{-(1-\alpha )s_{0}+\left(\frac{\text{1}}{\varphi^{\prime }}-\frac{\xi }{\delta }\right)\delta }$$ (48)

It can be seen from equation (46) and (48): because 0 < *α* < 1, the smaller , the larger n^*^. In order to satisfy that the n^*^ formula can be applied when *s*_*k*_ takes any value, the maximum value of n^*^ is taken:


$$n^{*}=\log_{\alpha }\left(\frac{\text{1}}{\frac{\left(1-\alpha )|s_{0}\right|}{\left(\frac{\text{1}}{\varphi^{\prime }}-\frac{\xi }{\delta }\right)\delta }+1}\right)$$ (49)

Therefore, the maximum number of steps required to enter the sliding mode belt is $N^{*}=1+\left\lfloor n^{*}\right\rfloor$. Because $\varphi^{\prime }$∈ (0, 1), the value of $\left\lfloor n^{*}\right\rfloor$ is:


$$\left\lfloor n^{*}\right\rfloor =\log_{\alpha }\left(\frac{\text{1}}{\frac{\left(1-\alpha )|s_{0}\right|}{\left(1-\frac{\xi }{\delta }\right)\delta }+1}\right)$$ (50)

*Remark* 5: under the discrete adaptive sliding mode control with error compensation, the sliding mode band width of the system is in the range of *O*(*T*^3^), and the control accuracy has been further improved.

## 4. Simulation and experiment

In this paper, the single-phase full bridge inverter circuit is equivalent to a buck circuit, the target waveform of the buck circuit is set as a sine wave, the output waveform is controlled to track the given sine wave, and the DC/AC inverter output control is realized. In order to verify the rationality of the proposed control algorithm, the simulation and experimental tests are carried out respectively when the given target values are constant signal, step signal, sinusoidal signal, etc. The parameters in the system are δ = 30, γ = 0.7, l = 1, c1 = 1000, α = 0.25, h = 5 × 10-4s respectively. During the simulation and experiment, the key components in the circuit in Fig 1 adopt the same parameters, as shown in Table 1.

**Table 1:** Circuit parameters configuration.

*Description*	*Parameters*	Nominal Value
*Input voltage*	*E*	80 *V*
*Inductance*	*L*	680*uH*
*Capacitance*	*C*	47*uF*
*Switching frequency*	*fs*	20 *kHz*
*Load resistance*	*R*	42 Ω/21 Ω

To ensure the reliability of the experimental results and the fairness of comparison, the parameters of the three controllers were tuned so that their key time-domain performance indices (rise time, settling time, overshoot, and steady-state error) reached comparable levels, as summarized in Table 2. It can be observed that all three controllers achieved response times shorter than 5 ms, overshoot below 1%, and nearly zero steady-state error. These results indicate that the controllers are comparable in terms of basic dynamic performance, thereby providing a fair basis for the subsequent performance analysis.

**Table 2:** Comparison of key performance indices of three controllers.

Performance Index	Traditional SMC Control	Adaptive Reaching Law DSMC	Improved Adaptive Reaching Law DSMC
Rise time (s)	0.0023	0.0022	0.0020
Settling time (s)	0.0044	0.0040	0.0040
Overshoot (%)	0.5292	0.2643	0.2102
Steady-state error (V)	40.2117	40.1057	40.0841

### 4.1. Simulation analysis

(1) Tracking test of step signal

**Fig 6:**
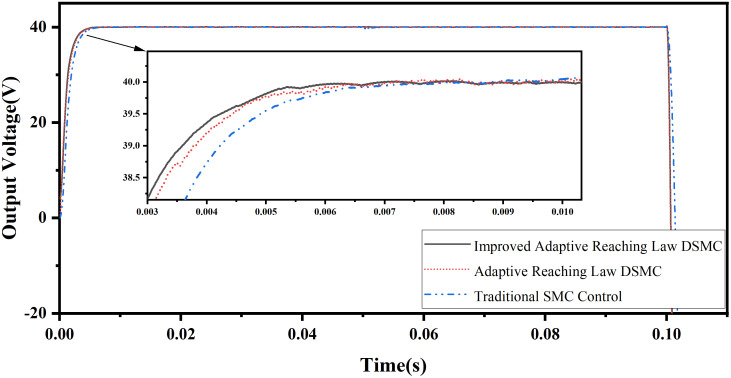
Load voltage waveforms of three controllers when the target value is a step signal.


The load is constant as *R* = 42 Ω, the given step target signal is shown in Equation (51), and the system output voltage waveform is shown in Fig 6.

$$v_{r}=\left\{ \begin{array}{c} 0V,fort<0\\ 40V,fort\geq 0 \end{array}\right.$$ (51)

The simulation results show that when the constant load is 42 Ω, the adaptive sliding mode control algorithm based on error compensation shows faster response speed and more accurate voltage tracking effect, and the tracking error is less than 0.1V.

From Equations (21), (29) and (37), it can be seen that the magnitude of the sliding mode band width of the traditional sliding mode control, adaptive sliding mode control and adaptive sliding mode control based on error compensation are $\frac{Td}{R_{o}C},\frac{\delta }{\varphi }+Cd_{k}^{*}$ and $\frac{\delta }{\varphi }+\xi$ respectively. The width of the sliding mode band is $\frac{Td}{R_{o}C}>\frac{\delta }{\varphi }+Cd_{k}^{*}>\frac{\delta }{\varphi }+\xi$, which shows that the adaptive sliding mode control based on error compensation has high accuracy.

(2) Voltage output simulation under load disturbance

**Fig 7:**
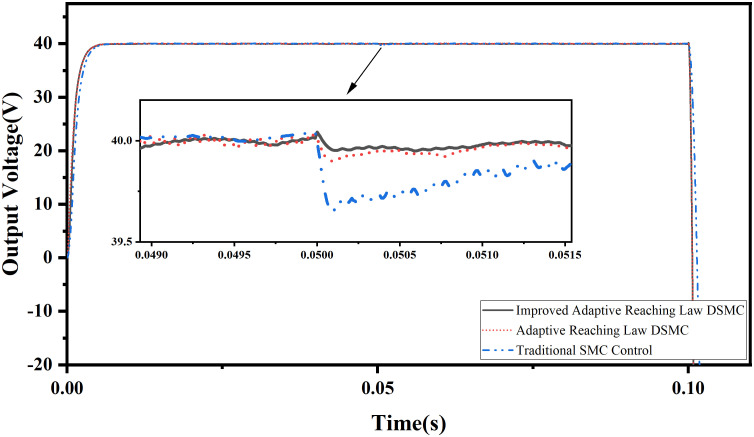
Tracking waveform of output voltage at sudden load change.

At 0.05s, switch the load from 42 Ω to 21 Ω, and the output voltage waveforms of the three control algorithms are shown in Fig 7. When the load current increases suddenly, the DC output voltage drops instantaneously, and the output voltage drop of the traditional sliding mode control is larger. Adaptive discrete sliding mode control and adaptive discrete sliding mode control based on error compensation have good anti disturbance characteristics. Because the adaptive discrete sliding mode control based on error compensation estimates the error and adds the external disturbance to the controller, its disturbance peak is lower than that of the traditional adaptive discrete sliding mode control.

(3) Simulation analysis of sliding mode strip width

**Fig 8:**
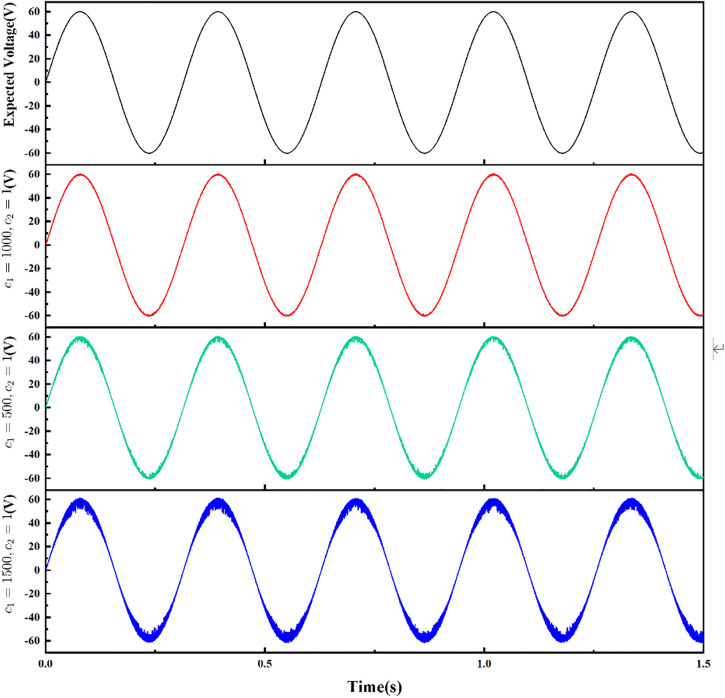
Simulation waveform of constant load sliding mode band.

Set the load *R* = 42 Ω, observe the sliding mode band width of adaptive discrete sliding mode control and adaptive discrete sliding mode control based on error compensation, as shown in Fig 8, the initial target value of the system is 40V, and set the target value of the output voltage of the system to -40v at 0.1s.

The simulation results show that the system quickly enters a stable state after a short jitter after startup. According to Equations (29) and (37), the sliding mode bandwidth of traditional adaptive sliding mode control and adaptive sliding mode control based on error compensation are Δ_1_ ≈ *δ* + *C*$d_{k}^{*}$ and Δ_2_ ≈ *δ* + *ξ* respectively. It can be seen from Fig 8 that the sliding mode bandwidth of the two algorithms is within this range. When the time reaches 0.1 s, the change of sliding mode band is caused by the negative half cycle voltage output by the inverter, and the system can quickly recover to a stable state.

(4) Tracking simulation of sine wave signal

**Fig 9:**
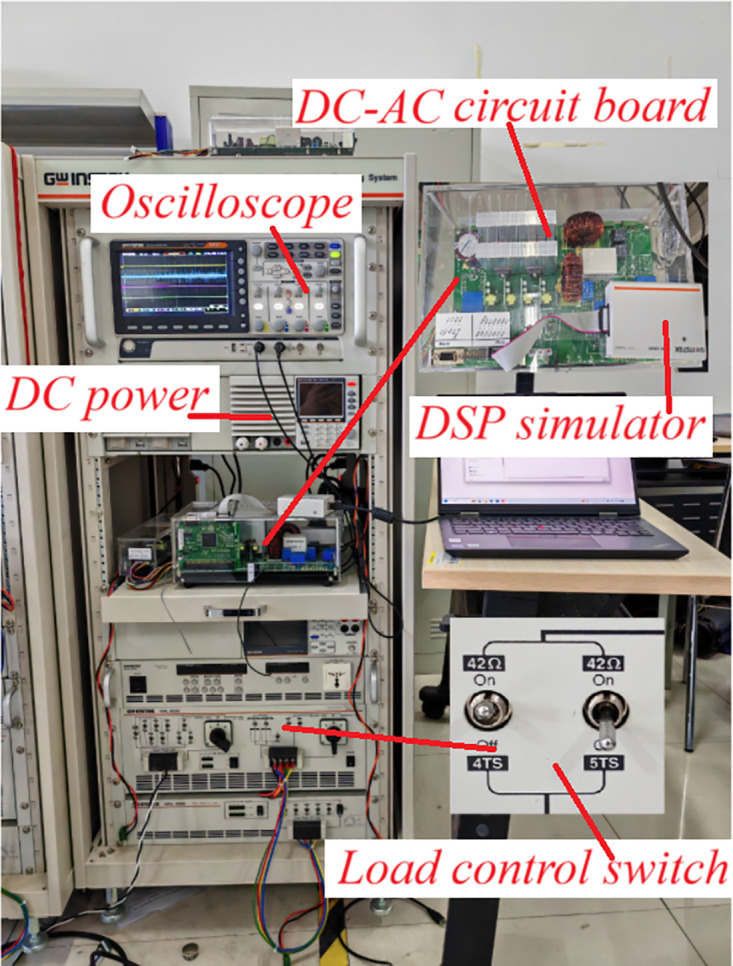
Sine wave output at different C matrices.

For the proposed adaptive sliding mode control algorithm based on error compensation, the output target value is set as the sine wave signal: *v*_r_=60sin(40πt). The influence of different parameter matrix C on the system response is discussed. The simulation results are shown in Fig 9. With the increase of the C matrix value, the response speed of the system increases, but the improper selection of parameters will affect the tracking effect of the system. Detailed analysis is as follows:

1) When *c*_1_ = 500, the response speed of the system is slow and the control gain is insufficient, resulting in a slight delay in the tracking process of the system and a decrease in the peak value of the output signal.
2) When *c*_1_ = 1500, although the response speed of the system is faster, the waveform is obviously thicker. The reason is that the larger control gain reduces the noise suppression ability of the system, resulting in the increased sensitivity of the system to high-frequency noise, thus amplifying the noise component in the waveform.
3) After many times of debugging, it is determined that under the conditions of *c*_1_ = 1000 and *c*_2_ = 1, the system can ensure the response speed, effectively suppress the noise, and the tracking effect is ideal.

### 4.2 Experimental test

**Fig 10:**
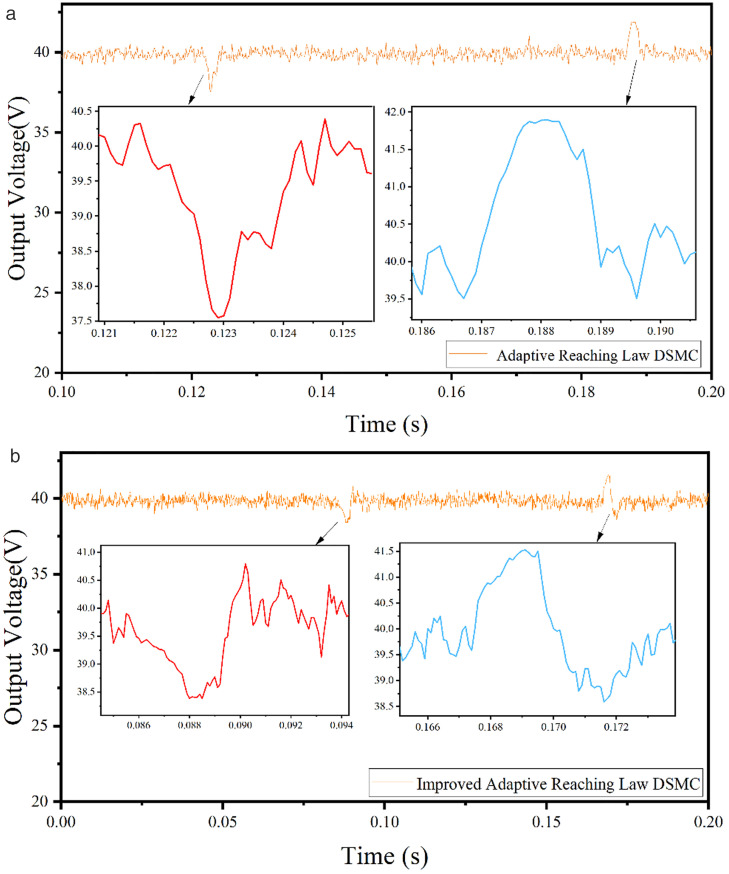
dc/ac experimental platform.

In order to verify the effectiveness of the designed control algorithm, actual tests were carried out on the experimental platform shown in Fig 10, and the parameters of each key device are the same as those in Table 1.

(1) Load disturbance test of DC output

**Fig 11:**
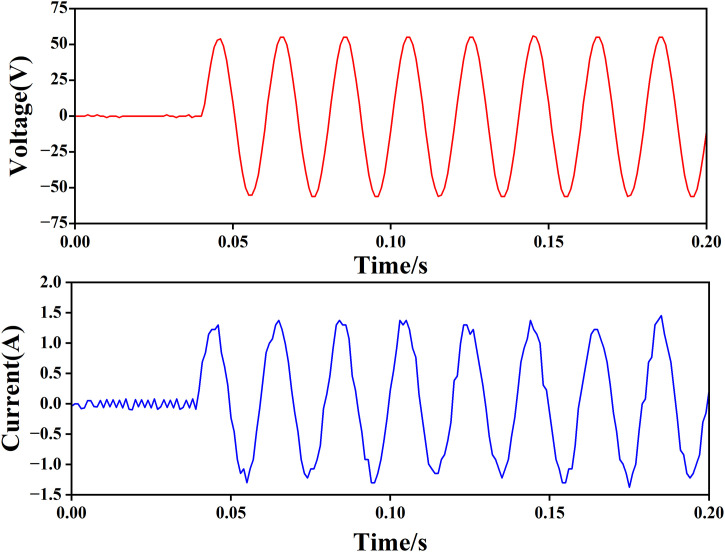
Response curves of two control algorithms to load disturbance. (a) Response curve of adaptive discrete sliding mode control algorithm to load disturbance. (b) Response curve of adaptive discrete sliding mode control algorithm based on error compensation to load disturbance.

The output target voltage is set to 40V, and the load resistance is switched between 21 Ω and 42 Ω. The adaptive discrete sliding mode control and the adaptive discrete sliding mode control algorithm based on error compensation are tested, as shown in Fig 11. Fig 11a shows the load voltage waveform of adaptive discrete sliding mode control. The left curve shows the voltage waveform when the load resistance is switched from 42 Ω to 21 Ω. The voltage drops suddenly, and the error is about 6%; The left curve is the voltage waveform when the load resistance is switched from 21 Ω to 42 Ω. The voltage increases suddenly, and the error is about 4.5%. Fig 11b shows the load voltage waveform of adaptive discrete sliding mode control based on error compensation. The left curve shows the voltage waveform when the load resistance is switched from 42 Ω to 21 Ω, and the voltage drops suddenly, with an error of about 3%. The right curve shows the voltage waveform when the load resistance is switched from 21 Ω to 42 Ω, and the voltage rises suddenly, with an error of about 3%. Compared with the two control algorithms, the adaptive discrete sliding mode control based on error compensation has better anti-interference ability.

(2) Sine wave output voltage test

The adaptive discrete sliding mode control algorithm based on error compensation is used to control the output voltage to sine wave, and the anti-interference performance of the system is verified by load switching.

**Fig 12:**
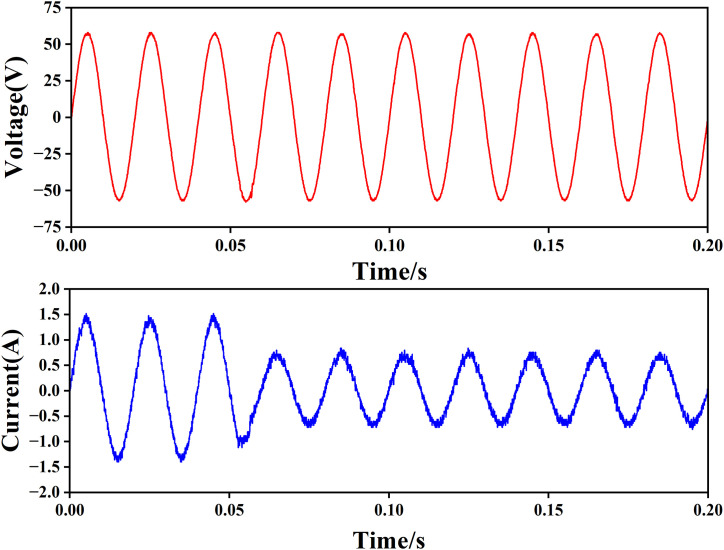
Voltage current output waveform when the load is 42 Ω.

**Fig 13:**
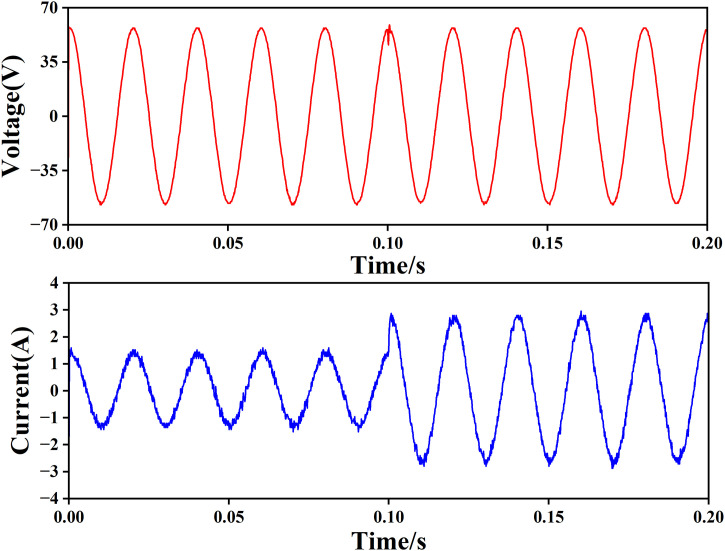
Voltage and current waveforms when the load resistance is switched from 21 Ω to 42 Ω.

1) When the load resistance is 42 Ω, as shown in Fig 12, the upper waveform is the sine wave output voltage with a peak value of 56V, and the lower waveform is the load current. It can be seen that the system can track the output sine wave voltage more quickly without transition time.

2) As shown in Fig 13, when the load resistance is switched from 21 Ω to 42 Ω at 0.05s, the distortion of the upper voltage waveform is small, and the lower current waveform can quickly become half of the original after the load is halved; In Fig 13, the load is switched from 42 Ω to 21 Ω at 0.1s, the load voltage above fluctuates slightly, and the current below can quickly become twice the original. It shows that the system has strong anti-disturbance ability.

The above experiments show that the single-phase full bridge inverter circuit is equivalent to a double buck circuit, and the adaptive discrete sliding mode control algorithm based on error compensation can realize the inverter output, and has good tracking performance and anti-interference ability.

## Conclusion

In this paper, the single-phase full bridge inverter circuit is divided into two buck circuits with positive and negative output voltage respectively. The target waveform of the output voltage is set as a sine wave, and the output of single-phase AC is realized through the real-time tracking control of the waveform. On the basis of traditional discrete sliding mode control, a new adaptive approach rate is introduced, which can dynamically adjust the control gain according to the distance between the sliding surface and the sliding band. When the state variable is far from the sliding surface, it accelerates the approach speed, and when the state variable approaches the sliding surface, it reduces the approach speed, which can effectively reduce chattering. As a result, the width level of the sliding mode band is reduced from the traditional *O(T*) to the same level *O(T*^3^), and the width of the sliding mode band is significantly reduced, significantly improving the control accuracy and jitter suppression ability. The feasibility of the proposed control strategy is verified by the research, simulation and experimental comparison of the traditional sliding mode control, discrete adaptive sliding mode control and discrete adaptive sliding mode control algorithm based on error compensation.

## Supporting information

S1 File Data availability statement. (ZIP)
